# Isatuximab for Stem Cell Transplant-Ineligible Multiple Myeloma: An Editorial

**DOI:** 10.7759/cureus.72177

**Published:** 2024-10-23

**Authors:** Abdul Haseeb Hasan, Muhammad Ali Abid, Bisma Tariq

**Affiliations:** 1 Medicine, Mayo Hospital, Lahore, PAK

**Keywords:** bortezomib, cd38, dexamethasone, fda, imroz, isatuximab, lenalidomide, monoclonal antibody, multiple myeloma

## Abstract

The recent approval of isatuximab in combination with bortezomib, lenalidomide, and dexamethasone (Isa-VRd) marks a significant advancement in the treatment of stem cell transplant-ineligible multiple myeloma (MM) patients. MM, the second most common hematological malignancy globally, poses substantial challenges, especially for patients who are unable to undergo autologous stem cell transplantation due to advanced age or comorbidities. Isatuximab, a CD38-targeting monoclonal antibody, has demonstrated promising efficacy in the pivotal trial, which reported a 40% reduction in disease progression or death for patients treated with Isa-VRd compared to bortezomib-lenalidomide-dexamethasone alone. The trial also showed higher rates of complete response and minimal residual disease (MRD)-negative status in the Isa-VRd group without introducing new safety concerns. These findings suggest that isatuximab’s integration into first-line therapy could substantially improve long-term outcomes for this underserved patient population. Clinicians are encouraged to adopt regular MRD monitoring and provide comprehensive patient education on potential side effects to optimize treatment adherence. Further research on isatuximab's use in combination with other emerging therapies may continue to expand therapeutic possibilities, offering hope for more effective management of MM.

## Editorial

Introduction

Multiple myeloma (MM) is currently the second most common blood cancer globally, characterized by a high degree of morbidity and mortality [1]. Although substantial strides have been made in the treatment landscape, a notable challenge remains. Patients who are ineligible for autologous stem cell transplantation (ASCT) experience decreased survival rates due to the limitations of available therapies [2]. The inability to undergo ASCT often stems from advanced age or the presence of comorbidities, which necessitates less aggressive treatment regimens [2].

One of the pivotal aspects of improving outcomes in MM is the achievement of minimal residual disease (MRD)-negative status, particularly in newly diagnosed MM [2]. Studies have demonstrated that patients who reach MRD-negative status tend to have significantly better overall survival and progression-free survival (PFS) [2]. Achieving MRD-negative status can serve as an early marker for favorable prognoses, guiding treatment strategies and decisions. The development of immunophenotypic and molecular techniques, including next-generation flow cytometry (NGF) and next-generation sequencing (NGS), has enhanced the precision of MRD detection, enabling clinicians to monitor residual disease with heightened accuracy [2]. These advances have led to the International Myeloma Working Group (IMWG) setting new guidelines for response assessment, further standardizing the evaluation of treatment effectiveness [2].

VRd and Rd regimen

The introduction of the bortezomib-lenalidomide-dexamethasone (VRd) regimen has been a cornerstone in the treatment of newly diagnosed MM patients who are ineligible for ASCT [2]. This combination therapy has become the standard of care, demonstrating a significantly improved overall survival compared to the lenalidomide-dexamethasone (Rd) regimen alone [2]. However, the growing recognition that novel agents, particularly monoclonal antibodies such as isatuximab, can be added to frontline treatments to achieve deeper and more durable responses has been an exciting development.

Isatuximab

Isatuximab is an IgG1 monoclonal antibody that targets CD38, a transmembrane protein highly expressed in MM cells [1-3]. Upon binding to CD38, isatuximab triggers multiple cytotoxic pathways, including complement-dependent cytotoxicity, antibody-dependent cellular cytotoxicity (ADCC), and antibody-dependent cellular phagocytosis [1,3]. ADCC, the dominant mechanism, enhances the activity of natural killer cells, promoting tumor cell destruction [3]. Additionally, isatuximab can directly induce apoptosis in MM cells, further reducing the disease burden [1,3]. These mechanisms collectively contribute to deeper treatment responses and higher rates of MRD-negative status in patients, which is associated with improved PFS and overall outcomes [1-3]. The drug has shown promise across several clinical trials, including the Phase 3 ICARIA-MM and IKEMA studies, where it was approved for use in combination therapies in multiple countries for relapsed and refractory MM [1-3].

FDA approval

The recent FDA approval of isatuximab with bortezomib, lenalidomide, and dexamethasone for treating stem cell transplant-ineligible MM patients was based on findings from the IMROZ trial [4]. The trial showed that treatment with isatuximab not only prolonged PFS but also higher rates of complete response and MRD-negative status [5]. It presents a new, highly effective option for a subset of patients who have traditionally faced limited treatment choices. This approval is likely to shift the standard of care and expand the horizons for MM treatment strategies, with isatuximab playing a central role in improving outcomes for transplant-ineligible patients.

IMROZ trial findings

The pivotal IMROZ trial sought to explore the efficacy of isatuximab in patients with newly diagnosed MM who were ineligible for ASCT [5]. This open-label, randomized Phase 3 trial enrolled 446 patients aged 18 to 80 years, who were randomly assigned to receive either the isatuximab-bortezomib-lenalidomide-dexamethasone (Isa-VRd) regimen or the VRd regimen alone. The primary endpoint of the study was PFS, which was evaluated according to IMWG criteria and assessed by an independent review committee. The patients in the Isa-VRd arm demonstrated a 40% reduction in the risk of disease progression or death. At the time of the analysis, the median PFS had not yet been reached in the Isa-VRd group, while the VRd group had a median PFS of 54.3 months. This improvement in PFS was significant, with a p-value of 0.0009, clearly indicating the superiority of the isatuximab-containing regimen​ [5].

Secondary endpoints of the trial further bolstered the case for isatuximab’s use in this patient population. The percentage of patients who achieved a complete response or better was notably higher in the Isa-VRd group (74.7%) compared to the VRd group (64.1%). Additionally, MRD-negative status was attained by a significantly larger proportion of patients in the Isa-VRd group, underscoring the depth of response elicited by adding isatuximab to the standard therapy [4,5]. Importantly, no new safety concerns were identified with the Isa-VRd regimen, and the incidence of serious adverse events, as well as adverse events leading to discontinuation, was comparable between the two groups. The most common adverse reactions observed in the study were musculoskeletal pain, diarrhea, upper respiratory tract infection, peripheral sensory neuropathy, rash, pneumonia, infusion-related reactions, insomnia, cataracts, fatigue, constipation, and peripheral edema [4,5].

Recommendations

To fully leverage the benefits of isatuximab, clinicians should focus on integrating it into first-line therapy, particularly for those unable to undergo ASCT due to age or other health conditions. Regular MRD monitoring using advanced technologies, like NGF and NGS, is essential to guide treatment strategies and optimize long-term results. Educating patients about the potential side effects, such as respiratory infections and infusion-related reactions, is also crucial for maintaining adherence and ensuring prompt management of adverse events. Finally, ongoing research into combining isatuximab with other innovative therapies will be critical in expanding treatment possibilities and further enhancing outcomes for this challenging patient population.
